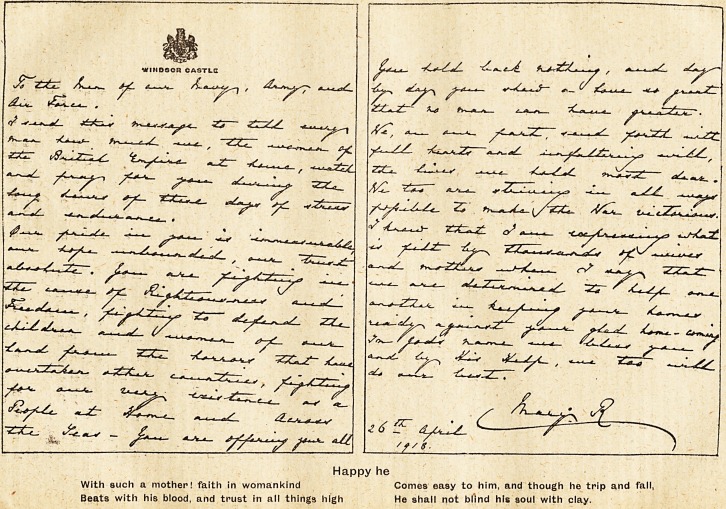# H M. Queen Mary, British Mothers, and Their Warrior Sons

**Published:** 1918-05-04

**Authors:** 


### H.M. QUEEN MARY, BRITISH MOTHERS, AND THEIR WARRIOR SONS.

Happy he
With such a mother! faith in womankind
Beats with his blood, and trust in all things high
Comes easy to him, and though he trip and fall,
He shall not blind his soul with clay.

## Figures and Tables

**Figure f1:**